# Breed-specific physiological, biochemical, and tissue transcript responses of Harri and Naemi lambs to acute heat stress

**DOI:** 10.3389/fvets.2026.1901604

**Published:** 2026-09-10

**Authors:** Nasif I. Abbas, Ahmed A. Al-Haidary, Ibrahim A. Alhidary, Hani H. Al-Baadani, Emad M. Samara, Mohammed A. Al-Badwi, Khalid A. Abdoun, Isiaka O. Olarinre, Abdulrahman S. Alharthi

**Affiliations:** Department of Animal Production, College of Food and Agriculture Sciences, King Saud University, Riyadh, Saudi Arabia

**Keywords:** heat stress, lambs, metabolism, mRNA expression, physiological

## Abstract

Climate change is an increasing threat to livestock production in arid regions. Understanding how indigenous breeds deploy immediate acute physiological and biochemical adjustments to acute hyperthermia at systemic and transcript-level indices is necessary to isolate initial homeostatic limits. This research compared the short-term thermoregulatory, metabolic, and multi-tissue transcriptomic adjustments of Naemi and Harri lambs exposed to acute heat stress. Forty male lambs (Naemi, Harri) were subjected to a 180-min exposure to either thermoneutral (25.0 °C Ta, 38.0% RH) or severe acute heat-stress (50.0 °C Ta, 13.2% RH) conditions in a controlled environmental chamber using a repeated-measures mixed-model approach. Thermophysiological kinetics, serum biochemistry, and relative mRNA expression of protective and metabolic genes (*HSP70*, *HSP90*, *PKM2*, *SCARB1*, *FGA*) in hepatic, skeletal muscle, and adipose tissues were evaluated after exposure. Acute heat stress induced a severe hyperthermic challenge without animal mortality. Harri lambs prioritized homeothermic defense through an intense, energy-demanding respiratory cooling response (panting). This active effort was accompanied by increased circulating glucose, triglycerides, and β-hydroxybutyrate, along with a concurrent increase in hepatic and muscle *SCARB1* transcript abundance. In contrast, Naemi lambs showed a distinct homeostatic buffering response through transient heterothermy and elevated baseline mRNA expression levels of the heat shock protein *HSP90* in hepatic tissue and *HSP70* in muscle tissue. Additionally, Naemi lambs showed a substantial, tissue-specific increase in *FGA* transcripts in subcutaneous adipose tissue, reflecting a localized transcriptional response within the fat tail matrix. These findings provide an integrated view of acute heat-stress coping patterns in two indigenous Saudi sheep breeds and provide exploratory target pathways. Because behavioral patterns, endocrine stress pathways, oxidative stress indicators, and functional protein validations were not performed, these findings represent macro-physiological and transcript-level profiles rather than a comprehensive welfare assessment or definitive functional validation.

## Highlights

- Acute heat stress exposure (50.0 °C) effectively differentiates the immediate coping mechanisms of desert-indigenous lamb breeds without causing lethal effects.
- Harri lambs employ an energy-intensive homeothermic defense, increasing panting while showing parallel increases in circulating lipids and tissue *SCARB1* transcripts.
- Naemi lambs demonstrate a distinct homeostatic buffering strategy through transient heterothermy and elevated baseline expression of the heat shock proteins *HSP70* and *HSP90*.
- A novel, tissue-specific surge in *FGA* transcripts was identified in the subcutaneous adipose tissue of Naemi lambs, pointing to localized transcriptional mobilization in the fat tail.

## 1 Introduction

Heat stress is a widespread and growing challenge to global livestock production, severely compromising animal health, welfare, and economic productivity (1). Driven by anthropogenic climate change, the frequency, duration, and intensity of extreme thermal events are projected to increase (2). This trend poses a significant threat to small ruminants, particularly lambs, which often represent the primary livestock sector in marginal regions but remain highly susceptible to thermal challenges because of their distinct physiological, metabolic, and anatomical characteristics (3). Because lambs play a critical role in global agriculture by supporting food security and stabilizing rural economies in vulnerable areas, understanding their acute homeostatic responses under extreme heat is essential to ensuring sustainable livestock production systems (4). When exposed to elevated ambient temperatures, small ruminants initiate a complex, multilayered array of physiological, biochemical, and cellular adjustments to maintain homeostatic equilibrium (5).

Immediate, reflex-driven physiological responses include peripheral vasodilation to maximize sensible heat dissipation and increased respiration rates (panting) to enhance respiratory evaporative cooling (6). Simultaneously, severe thermal stress causes significant shifts in energy metabolism (7). Animals typically experience reduced voluntary dry matter intake, prompting a metabolic shift from conventional carbohydrate oxidation to rapid lipid mobilization and altered protein catabolism to meet the energetic demands of thermoregulation (8). The efficiency, energetic costs, and specific phenotypic responses used during thermal challenges vary significantly among breeds (9). This variation reflects localized genetic predispositions and unique historical environmental backgrounds. For example, some indigenous breeds thrive in hyper-arid zones by using transient heterothermy – an adaptation in which the animal temporarily tolerates an elevated core body temperature during peak thermal periods to conserve vital water and energy that would otherwise be spent on evaporative cooling (10). In contrast, other breeds rely on immediate, energy-intensive homeothermic defense mechanisms, such as intense tachypnea, which aggressively maintains core temperature stability but imposes a heavy systemic metabolic cost (11).

At the cellular level, these macrophysiological adjustments are accompanied by a complex network of transcriptional changes. Exposure to hyperthermia triggers a widespread heat shock response, primarily characterized by the rapid accumulation of transcripts encoding heat shock proteins 70 (*HSP70*) and 90 (*HSP90*), which are integral components of cellular proteostasis networks (12). Simultaneously, tissue-specific metabolic transcripts– including pyruvate kinase M2 (*PKM2*), which regulates glycolytic pathways, and scavenger receptor class B member 1 (*SCARB1*), which modulates lipid transport – are differentially regulated in parallel with systemic energy shifts (13, 14). Although the general physiological and metabolic effects of heat stress on lambs have been widely documented (1, 15), comprehensive, multidimensional comparative analyses directly linking systemic thermo-physiological trade-offs to biochemical changes and tissue-specific transcriptomic responses in desert-adapted breeds remain limited.

Naemi and Harri lambs are two socioeconomically important fat-tailed breeds native to the Arabian Peninsula (16). Although both have evolved under extreme arid climates characterized by prolonged hyperthermia and intense solar radiation, empirical observations indicate that they display distinct phenotypic behaviors and thermoregulatory thresholds. We hypothesize that Harri and Naemi lambs deploy divergent physiological coping strategies under acute hyperthermic strain, involving distinct metabolic and transcriptomic trade-offs. Specifically, Harri lambs rely primarily on an immediate, high-cost homeothermic defense driven by intense respiratory cooling, resulting in pronounced depletion of circulating energy substrates and elevated systemic metabolic strain. Conversely, Naemi lambs are expected to employ a resource-conserving strategy characterized by transient heterothermy, moderated metabolic adjustments, and baseline heat-shock transcriptional readiness (HSP70, HSP90), allowing homeostatic buffering without immediate, high-cost systemic expenditure.

This research aims to elucidate the physiological, biochemical, and transcriptional trade-offs underlying acute heat stress coping mechanisms in Naemi and Harri lamb breeds. By continuously monitoring microclimatic indices, tracking thermophysiological kinetics, quantifying blood metabolic profiles, and measuring the relative mRNA expression of target protective and metabolic genes in animals exposed to a controlled acute hyperthermic challenge, we sought to isolate and comprehensively map their divergent, tissue-specific molecular transcriptional coping strategies. This high-intensity, short-term model was specifically selected to isolate immediate physiological tipping points and establish an exploratory baseline of multi-tissue transcript-level responses prior to future functional proteomic validation.

## 2 Materials and methods

### 2.1 Ethical statement and experimental site

All experimental protocols, handling procedures, blood sampling, and surgical biopsy interventions were reviewed and formally approved by the Institutional Animal Care and Use Committee of King Saud University, Riyadh, Saudi Arabia (Approval No. KSU-IACUC-24-9), in accordance with institutional and international guidelines for biomedical research involving animals. The research was conducted in the climate-controlled research facility of the Experimental Station, Department of Animal Production, College of Food and Agricultural Sciences, King Saud University, Riyadh, Saudi Arabia (latitude: 24°48′N; longitude: 46°31′E). The surrounding geographic has a hyper-arid climate, with summer ambient temperatures frequently exceeding 45 °C. Accordingly, the experimental heat-stress protocol was designed as a controlled, short-term severe hyperthermic challenge relevant to extreme thermal episodes in arid production environments, while recognizing that chamber exposure does not fully reproduce all features of commercial field conditions.

### 2.2 Animal, housing, and diet

Forty healthy male lambs, including 20 Naemi and 20 Harri, aged 3 months with an initial mean body weight of 22.5 ± 1.0 kg, were selected for this research. Before the experiment began, all animals underwent a thorough veterinary examination to ensure they were free from clinical disease, internal parasites, and external vector infestations. The animals were housed individually in dedicated metabolism pens (2 m high × 1.5 m wide) within a climate-controlled facility.

Each pen was equipped with an automated individual feeding trough to monitor feed intake. Throughout the research, the lambs had ad libitum access to fresh water and a standardized diet. The diet was formulated as a complete pelleted feed to meet or exceed National Research Council (17) requirements for growing small ruminants, as shown in Table 1.

**Table 1:** Ingredients and nutrient composition of pelleted complete feed offered to lambs during the experiment.

Item	Composition
Ingredients (as feed, %)	
Barley grain	30.45
Wheat feed	26.00
Wheat bran	5.00
Palm kernel cake	17.55
Soya hulls	13.40
Salt	1.00
Limestone	2.50
Molasses	3.00
Acid buffer	1.00
Commercial premix^*^	0.10
Composition (as DM, %)	
DM, %	90.8
CP, %	13.24
CF, %	12.72
ME, Mcal/kg	2.79
Ca, %	1.90
P, %	0.39
Fe, μg/g	193
Cu, μg/g	12.4
Zn, μg/g	39.3
Mn, μg/g	133
Se, μg/g	0.48

* Commercial premix composition per kg: vitamin A (10,000 IU), vitamin D (1,000 IU), vitamin E (20 IU), magnesium (300 mg), copper (24 mg), cobalt (0.6 mg), iodine (1.2 mg), manganese (60 mg), selenium (0.3 mg), zinc (60 mg). ^2^Values are expressed on dry matter (DM) basis ^3^Mineral concentrations are expressed in their respective units as shown (%, μg/g) ^4^ME, metabolizable energy.

### 2.3 Experimental design and environmental chamber protocol

The research consisted of two chronological stages: a 2-week baseline adaptation phase followed by a 6-week intensive experimental evaluation phase. The 6-week period was used to sequentially block, habituate, and expose individual animal cohorts to standardized, acute environmental challenges, thereby minimizing the confounding influence of seasonal or uncontrolled diurnal microclimatic variation. During acute testing events, animals were tested in simulated environmental pens equipped with automated thermodynamic programming sensors.

The experiment used a sequential repeated-measures design in which each lamb was evaluated under both target thermal microclimates: thermoneutral conditions and acute heat-stress conditions. Thermoneutral exposure was conducted first, followed by a standardized 7-day washout and homeostatic recovery period under constant thermoneutral conditions, after which the same lambs were exposed to the acute heat-stress condition. This structure allowed each lamb to serve as its own repeated experimental unit for comparisons between environmental conditions. Because the exposure sequence was uniform and nonrandomized across all animals due to strict institutional chamber allocation logs, this layout is classified as a sequential repeated-measures model rather than a randomized, counterbalanced crossover design. To account for potential carryover effects or chronological biases, baseline physiological checks were strictly performed before the second challenge to verify that all lambs had returned to post-washout homeostatic baselines. Nevertheless, because all animals completed the environmental conditions in an identical chronological order, standalone temperature effects cannot be fully disentangled from potential confounding factors, including prior chamber habituation, secondary human handling, and cellular healing responses stemming from the initial biopsy procedure. Causal interpretations regarding environmental temperature variations are constrained by this fixed-order sequence.

For the thermoneutral condition, all pens for each breed inside the chamber were maintained at a steady baseline temperature of 25.0 °C and a relative humidity of approximately 38.0%. For the acute heat-stress condition, chamber temperature was abruptly elevated to 50.0 °C, and relative humidity was adjusted to approximately 13.2%. For each assigned microclimate, lambs of each breed were kept inside the chambers under the programmed environmental target for exactly 180 min.

Continuous air velocity within the chamber was fixed at a minimal baseline (< 0.2 m/s) to eliminate convective cooling as a confounding variable, and a standardized 12:12-h light–dark photoperiod was enforced. To reduce handling stress or physical shock caused by new management procedures, the animals were thoroughly desensitized to human contact and chamber entry protocols during the initial 2-week adaptation period. The experimental trial ended after all individual lambs from the Naemi (*n* = 20) and Harri (*n* = 20) breeds successfully completed their full 180-min exposure sequences under both distinct environmental conditions. Immediate post-exposure intervals were strictly reserved for thermophysiological profiling, blood sampling, and tissue biopsy collection.

### 2.4 Meteorological measurements

To continuously mapping the thermodynamic profiles of the experimental environments, three high-precision automated data loggers (TW-USB-2-LCD+, ThermoWorks Inc., Lindon, UT, United States) were deployed within the simulation chambers. The loggers were suspended directly above the environmental pens at a standardized height of approximately 2.0 m to measure ambient microclimatic conditions immediately above the animals’ breathing zone, while avoiding convective interference from body heat or direct animal contact. The units were programmed to continuously sample and record ambient temperature (Ta, °C) and relative humidity (RH, %) at synchronized 30-min intervals throughout both the thermoneutral and acute heat stress exposure periods (18).

Proprietary software (ThermoData Standard Utility, ThermoWorks Inc., United States) was used to configure the recording parameters, synchronize the hardware internal clocks, and securely download the raw environmental datasets. To quantify the cumulative thermal burden experienced by the Naemi and Harri lambs, the raw Ta and RH data points were used to calculate the temperature-humidity index (THI). The mathematical model used was the modified equation proposed by Marai et al. (19), which is specifically calibrated for small ruminants under arid and semi-arid environmental conditions:

$$\mathrm{THI}=(\mathrm{Ta}-0.31)-(0.31x\frac{\mathrm{RH}}{100}\mathrm{xTa})X14.4$$

Where Ta represents the recorded ambient air dry-bulb temperature (°C). RH represents the recorded relative humidity as a whole percentage (%). The resulting calculated THI values were categorized into recognized bioclimatic stress thresholds for small ruminants: THI < 70 (absence of Stress), 70 < THI < 75 (moderate heat stress), 75 < THI < 82 (severe heat stress), and THI > 82 (extreme/extreme severe heat stress) according to Nicolás-López et al. (20). These calculations directly confirmed that the microclimates maintained within the chambers corresponded to a stable, comfortable baseline during the thermoneutral phase and shifted to an acute, extreme thermal state during the 180-min 50 °C HS challenge phase.

### 2.5 Thermophysiological measurements

At the end of each standardized 180-min environmental exposure block (under both the 25.0 °C thermoneutral and 50.0 °C acute heat stress microclimates), an immediate, intensive thermophysiological profile was recorded for each lamb (*n* = 40) according to the previously described (20, 21). All physiological data were collected twice in succession within a 5-min window by the same technical operator to eliminate inter-observer variance, and the mean values were used for downstream analysis.

Rectal temperature (Tr, °C) was measured to quantify true internal core body temperature. A high-precision digital thermistor thermometer (Traceable™ Digital Thermometer, Control Company, Friendswood, TX, United States) equipped with a flexible, lubricated vinyl-coated probe was inserted atraumatically into the rectum to a standardized depth of 7.0 cm. The probe was maintained in direct contact with the mucosal wall until a stable electronic equilibrium plateau was reached.

Skin (Tsk, °C) and coat (Tcoat, °C) temperatures were measured using a calibrated non-contact infrared thermometer (Traceable™ Mini IR Thermometer, Control Company, Friendswood, TX, United States) with an emissivity factor set to 0.98, appropriate for livestock tissue. Skin temperature (Tsk) was obtained by holding the infrared lens perpendicular at exactly 15.0 cm from closely shaved anatomical regions at the left shoulder and hip. Outer fleece/coat surface temperature (Tcoat) was recorded from the same anatomical regions before shaving to assess the thermal insulating boundary layer.

Respiratory rate (RR, breaths/min) was determined by direct auscultation with a clinical Littmann stethoscope placed over the thoracic cavity between the 9th and 11th intercostal spaces. To prevent hyperventilation artifacts from handling, the operator stood quietly outside the lamb’s immediate flight zone. The exact time in seconds required for the animal to complete 10 fully realized respiratory cycles (inspiration and expiration) was measured with a digital stopwatch. This duration was then mathematically converted into standard physiological units using the following formula according to (22):

$$\mathrm{RR}\;(\text{breaths}/\min)=(\frac{10}{\text{Measured time in seconds}})X\;60$$

To combine these individual physiological traits into a unified index of heat tolerance, the Adaptability Coefficient (AC) was calculated for each animal in both thermal states. The mathematical framework used followed the validated index described by Pascual-Alonso et al. (21), which is based on Bresson’s baseline principles:

$$\mathrm{AC}=(\frac{\mathrm{Tr}}{39.1})X\;10+(\frac{\mathrm{RR}}{23})$$

Where Tr represents the measured internal rectal core temperature (°C) under the target condition. 39.1 °C is established as the ideal physiological core baseline constant for healthy small ruminants. RR represents the calculated respiratory rate (breaths/min) under the target condition. 23 breaths/min is established as the baseline ideal physiological respiration constant for non-stressed lambs. Under this indexing system, an AC value closest to 11.0 indicates perfect homeostatic stability and high thermal adaptability, while progressively higher values indicate severe homeostatic disruption and low heat tolerance.

### 2.6 Serum biochemical measurements

Immediately after each 180-min environmental exposure phase, whole blood samples (9 mL) were collected from each lamb via aseptic jugular venipuncture. In all animals, blood was drawn into plain clot-activator tubes for serum extraction. Serum was separated by centrifugation at 3,000 × g for 15 min at 4 °C. The resulting supernatant serum was aliquoted into sterile 1.5 mL microcentrifuge tubes and frozen at −20 °C for subsequent batch biochemical analysis to prevent inter-assay variation. Quantitative determination of serum total protein (TP), albumin (ALB), glucose (GLU), triglycerides (TRI), urea, creatinine (CRT), alanine aminotransferase (ALT), aspartate aminotransferase (AST), and β-hydroxybutyrate (BHB) concentrations was performed using spectrophotometric assays on a semi-automated clinical chemistry analyzer (RX Monza, Randox Laboratories Ltd., Crumlin, United Kingdom). All reactions were calibrated and validated with commercial diagnostic kits, reagents, and quality control standards provided by the manufacturer (Randox Laboratories, Antrim, United Kingdom).

### 2.7 Tissue biopsy and RT-qPCR analysis

Tissue biopsies were surgically collected from three primary metabolic compartments in all animals, hepatic tissue, skeletal muscle tissue, and subcutaneous adipose tissue, immediately after each 180-min environmental exposure phase under both 25 °C thermoneutral and 50 °C acute heat-stress conditions. Biopsy sites were prepared aseptically, and tissue collection was performed under local anesthesia. Animals were monitored until full recovery, with sites inspected for bleeding, swelling, or other complications; different sites within the same anatomical region were used at each sampling point to minimize the influence of healing from the first biopsy on the second.

Hepatic tissue was collected from the right side of the animals between the 9th and 11th intercostal ribs using a puncture biopsy approach. Skeletal muscle tissue was collected from the rear leg region near the tail, and subcutaneous adipose tissue was collected from the tail region. Tissue fragments of approximately 50 mg were immediately snap-frozen in liquid nitrogen and stored at −80 °C until total RNA extraction.

Total mRNA was isolated from frozen tissue using the QIAzol™ Lysis Reagent protocol (Qiagen Inc., Hilden, Germany), based on a modified acid guanidinium thiocyanate-phenol-chloroform extraction method. To eliminate potential genomic DNA (gDNA) contamination, the mRNA pellets were treated with an RNase-Free DNase I kit (Qiagen Inc., Hilden, Germany). The absolute concentration and purity of the resulting RNA eluates were determined by microvolume spectrophotometry using a NanoDrop™ ND-1000 Spectrophotometer (NanoDrop Technologies, Wilmington, DE, United States). Validated RNA samples were diluted to 100 ng/μL and used for first-strand cDNA synthesis in a 20 μL reaction.

Oligonucleotide primer pairs targeting specific *Ovis aries* transcript sequence templates were used to quantify the relative mRNA expression of five primary protective and metabolic genes: heat shock protein 70 (*HSP70*), heat shock protein 90 (*HSP90*), pyruvate kinase M2 (*PKM2*), scavenger receptor class B member 1 (*SCARB1*), and fibrinogen alpha chain (*FGA*), as shown in Table 2. All qPCR reactions were performed on an advanced real-time PCR platform (7,300 Real-Time PCR System, Applied Biosystems, United Kingdom) using a fluorescent SYBR™ Green Master Mix dye. All samples were analyzed in technical triplicate. The absolute cycle threshold (CT) values for each target were normalized to the geometric mean of validated stable endogenous reference gene expression (*β*-actin) to calculate ΔCT values. Relative mRNA expression between experimental thermal states and breeds was determined using the comparative 2^−ΔΔCT^ mathematical model as previously described (23). Because this approach quantifies transcript abundance only, molecular findings were interpreted strictly as messenger RNA-level responses and not as direct evidence of protein abundance, enzyme activity, or confirmed functional cellular protection.

**Table 2:** Target genes, primer sequences, and accession numbers used for RT-qPCR analysis in hepatic, skeletal muscle, and adipose tissues of Harri and Naemi lambs.

Genes^1^	Primer sequence	Accession number
*HSP70*	F: TGGCTTTCACCGATACCGAG R: TCGTTGATCACGCGGAAAG	NM_001285703.1
*HSP90*	F: AAGAGCCTGACCAACGACTG R: AAAGGAGCTCGTCTTGGGAC	XM_005695418.1
*PKM2*	F: CCCTGGAGAAGAGCTACGAG R: CTGTCTGGTGATTCCGGGTC	GCF_016772045.2
*SCARB1*	F: TCGCTGACATGGGCAACCTC R: GACAGGCTGTTGGGGTCGAT	XM_060401087.1
*FGA*	F: AGTCAGACCTACCCACGGC R: ACCGCCGACAGGATCACTA	XM_004017183.5
*β-actin*	F: CCTTCCTGGGTAGGTGTCG R: TGGCGTAGAGGTCCTTCCTG	AJ312193.1

1 *HSP70*, heat shock protein 70; *HSP90*, heat shock protein 90; *PKM2*, pyruvate kinase M2; *SCARB1*, scavenger receptor class B member 1; *FGA*, fibrinogen alpha chain. *β-actin* was used as the endogenous reference gene for normalization.

### 2.8 Statistical analysis

All statistical analyses were performed using SAS software version 9.4 (24). Before formal hypothesis testing, data were screened for completeness and potential outliers. Residual normality was assessed using the Shapiro–Wilk test, and homogeneity of variance was evaluated using Levene’s test with the appropriate SAS procedures. Diagnostic residual plots were inspected as needed to verify compliance with model assumptions.

Because the same lambs were evaluated under both thermoneutral and acute heat-stress conditions, observations were not treated as independent. Therefore, all animal-level physiological, biochemical, enzymatic, and transcript-level data were analyzed with a repeated-measures mixed-model approach using the PROC MIXED procedure in SAS. The least-squares means, standard errors, and *p*-values reported in all tables and figures were derived from this mixed-model analysis. Breed, environmental temperature, and their interaction were included as fixed effects. Individual lamb nested within breed was included as the subject effect to account for repeated observations from the same animal, and environmental temperature was treated as the repeated factor within the animal. The statistical model was expressed as follows:

Yijk=μ+Bi+Tj+(BxT)ij+AK(i)+εijk

Where Yijk represents the observed value of the dependent variable; μ denotes the overall mean; Bi denotes the fixed effect of the ith breed (i = Harri or Naemi); Tj denotes the fixed effect of the jth environmental temperature condition (j = thermoneutral or acute heat stress); (B × T)ij denotes the breed × temperature interaction; Ak(i) denotes the random effect of animal k nested within breed I; and εijk denotes the residual error term.

Because animals were exposed to the thermoneutral and heat-stress conditions in a fixed, non-randomized sequence rather than a randomized crossover, the breed × temperature interaction term is used to test whether the magnitude of the within-breed change from thermoneutral to heat-stress conditions differed between the Harri and Naemi breeds, rather than to draw independent causal inferences about breed or temperature effects considered alone. Accordingly, throughout the Results and Discussion, significant breed × temperature interactions are reported and interpreted as breed-dependent, within-breed before-after differences, and causal language describing these interactions has been minimized.

To verify the procedural validity of the sequential design and rule out chronological or carryover effects, the pre-second-exposure baseline physiological parameters were formally analyzed against the initial baseline values using a paired t-test (*p* > 0.05). Because these tests confirmed complete homeostatic recovery, only the standard baseline values were used in the main repeated-measures model.

Relative mRNA expression was analyzed separately in hepatic, skeletal muscle, and adipose tissues to preserve tissue-specific interpretation. Within each tissue, the same mixed-model structure was applied, with breed, temperature, and breed × temperature as fixed effects and animal nested within breed as the repeated subject. Least-squares means were generated for breed × temperature combinations, and pairwise comparisons were adjusted using Tukey’s test when the interaction term was significant.

Results are presented as least-squares means with pooled standard error of the mean. Statistical significance was declared at *p* < 0.05, and tendencies were considered when 0.05 ≤ *p* < 0.10. For compact reporting, *p*-values lower than 0.001 are reported as *p* < 0.001. Different superscript letters within the same row, panel, or variable indicate significant differences among breed × temperature least-squares means at *p* < 0.05. Given the number of physiological, biochemical, enzymatic, and transcript-level endpoints evaluated, the potential for inflated type I error due to multiple testing was considered when interpreting the results. Consequently, this multi-endpoint analysis is explicitly identified as exploratory and only nominal *p*-values (uncorrected for multiplicity) are reported throughout the Results and Tables.

## 3 Results

### 3.1 Meteorological profiles

The effects of environmental thermal treatments (thermoneutral and acute heat stress) on chamber microclimatic parameters and calculated thermodynamic indices monitored during the experimental periods are presented in Table 3. The results show that chamber ambient temperature (Ta), relative humidity (RH), and the resulting Temperature-Humidity Index (THI) during the thermoneutral control stage were influenced by the interaction between environmental thermal treatments (temperature) and breed (*p* < 0.05). Both lamb breeds (Harri and Naemi) had lower Ta and THI under thermoneutral conditions than under heat stress conditions (*p* = 0.001). In contrast, RH was higher in both breeds under thermoneutral conditions than under heat stress conditions (*p* = 0.046). This confirms that both Naemi and Harri lamb cohorts were uniformly exposed to a standardized, severe acute heat shock environment throughout their respective 180-min testing periods. The lamb breeds were not affected by environmental condition parameters (*p* > 0.05). However, there were significant differences between thermoneutral and heat stress conditions (*p* = 0.0001).

**Table 3:** Environmental conditions under thermoneutral and heat stress conditions in Naemi and Harri lambs.

Variables^1^		Ta (°C)	RH (%)	THI
Interaction (breed × temperature)				
Harri	25 °C	24.52^b^	36.77^a^	53.50^b^
	50 °C	51.33^a^	13.16^b^	93.09^a^
Naemi	25 °C	24.73^b^	39.22^a^	59.24^b^
	50 °C	50.97^a^	13.27^b^	93.26^a^
SEM^2^		0.92	1.56	6.74
*p*		0.001	0.046	0.001
Breed				
Harri		37.93	24.97	73.30
Naemi		37.85	26.25	76.25
SEM		0.65	1.10	4.76
*p*		0.936	0.419	0.664
Temperature				
25 °C		24.63^b^	38.00^a^	56.37^b^
50 °C		51.15^a^	13.22^b^	93.17^a^
SEM		0.65	1.10	4.76
*p*		0.0001	0.0001	0.0001

Different lowercase letters (a-b) within the same column indicate significant differences (*p* < 0.05) between temperature treatments or between breeds.

1 Variables: Ta, ambient temperature; RH, relative humidity; THI, temperature humidity index.

2 Values are expressed as means ± SEM.

### 3.2 Thermophysiological responses

The effects of environmental thermal treatments (thermoneutral and acute heat stress) on thermo-physiological responses and adaptability coefficient in Naemi and Harri lambs are presented in Table 4. The interaction between environmental thermal treatments (temperature) and breed had a significant effect on respiratory rate (RR), skin temperature (Tsk), coat temperature (Tct), and adaptability coefficient (AC; *p* < 0.05), while rectal temperature (Tr) was not affected (*p* > 0.05). Both Harri and Naemi lambs had higher RR, Tsk, Tct, and AC under heat stress than under thermoneutral conditions (*p* = 0.003, *p* = 0.045, *p* = 0.043, and *p* = 0.020, respectively). The lamb breeds were not affected by thermo-physiological kinetics and adaptability indices (*p* > 0.05). However, there were significant differences between thermoneutral and heat stress conditions (*p* = 0.0001). Additionally, lambs under heat stress (50 °C) had higher Tr than under thermoneutral conditions at 25 °C (*p* = 0.018).

**Table 4:** Thermo-physiological kinetics and adaptability indices under thermoneutral and heat stress conditions in Naemi and Harri lambs.

Variables^1^		RR	Tr	Tsk	Tct	AC
Interaction (breed × temperature)						
Harri	25 °C	41.27^b^	39.33	32.38^b^	25.97^c^	7.87^b^
	50 °C	120.34^a^	39.84	36.63^a^	39.29^b^	13.70^a^
Naemi	25 °C	50.28^b^	38.91	32.13^b^	25.36^c^	8.03^b^
	50 °C	117.50^a^	39.76	36.13^a^	43.71^a^	13.61^a^
SEM^2^		3.31	0.27	0.80	2.20	0.25
*p*		0.003	0.546	0.045	0.043	0.020
Breed						
Harri		80.81	39.33	34.51	32.63	10.82
Naemi		83.89	39.59	34.13	34.53	10.78
SEM		2.34	0.19	0.57	2.26	0.17
*p*		0.359	0.357	0.647	0.556	0.887
Temperature						
25 °C		45.78^b^	39.12^b^	32.26^b^	25.66^b^	7.95^b^
50 °C		118.92^a^	39.80^a^	36.38^a^	41.50^a^	13.65^a^
SEM		2.34	0.19	0.57	2.26	0.17
*p*		0.0001	0.018	0.0001	0.0001	0.0001

Different lowercase letters (a–c) within the same column indicate significant differences (*p* < 0.05) between temperature treatments or between breeds.

1 Variables: RR, respiratory rate (breaths/min); Tr, rectal temperature; Tsk, skin temperature; Tct, coat temperature; AC, adaptability coefficient.

2 Values are expressed as means ± SEM.

### 3.3 Serum biochemical and hepatic enzymatic responses

The effects of environmental thermal treatments (thermoneutral and acute heat stress) on serum biochemical and hepatic enzymatic parameters in Naemi and Harri lambs are presented in Table 5. The current results show that environmental thermal treatments (temperature) or interactions between temperature and breed had a significant effect (*p* < 0.05) on total protein (TP), albumin (ALB), and triglyceride (TRI) concentrations, while breed alone did not have a significant effect (*p* > 0.05).

**Table 5:** The serum biochemical and hepatic enzymatic responses under thermoneutral and heat stress conditions in Naemi and Harri lambs.

Variables^1^		TP (g/dl)	ALB (g/dl)	GLU (mg/dl)	TRI (mg/dl)	UREA (mg/dl)	CREA (mg/dl)	ALT (U/L)	AST (U/L)	BHB (mmol/l)
Interaction (breed × temperature)										
Harri	25 °C	5.03^b^	2.36^b^	71.06^b^	68.90^ab^	16.12^b^	1.71	27.10^a^	86.45	0.34
	50 °C	5.65^a^	2.71^a^	81.03^a^	73.23^a^	25.10^a^	1.85	28.25^a^	89.12	0.45
Naemi	25 °C	5.28^ab^	2.54^ab^	63.25^c^	63.73^b^	13.63^b^	1.72	25.45^b^	85.63	0.31
	50 °C	5.72^a^	2.69^a^	62.29^c^	72.33^a^	16.65^b^	1.77	25.29^b^	82.03	0.42
SEM^2^		0.14	0.05	2.19	1.90	1.16	0.05	0.69	1.67	0.01
*p*		0.043	0.046	0.017	0.026	0.015	0.060	0.035	0.069	0.635
Breed										
Harri		5.34	2.54	76.04^a^	71.06	20.61^a^	1.71	27.67^a^	87.78^a^	0.40^a^
Naemi		5.50	2.61	62.77^b^	68.03	15.14^b^	1.75	25.37^b^	83.83^b^	0.36^b^
SEM		0.10	0.03	1.55	1.34	0.82	0.04	0.48	1.18	0.01
*p*		0.289	0.133	0.0001	0.119	0.0001	0.565	0.002	0.024	0.003
Temperature										
25 °C		5.16^b^	2.45^b^	67.15^b^	66.32^b^	14.88^b^	1.72	26.27	86.04	0.32^b^
50 °C		5.69^a^	2.71^a^	71.66^a^	72.78^a^	20.88^a^	1.81	26.77	85.57	0.43^a^
SEM		0.11	0.03	1.55	1.34	0.82	0.04	0.48	1.18	0.01
*p*		0.001	0.0001	0.047	0.001	0.0001	0.070	0.479	0.783	0.0001

Different lowercase letters (a–c) within the same column indicate significant differences (*p* < 0.05) between temperature treatments or between breeds.

1 Variables: TP, total protein; ALB, albumin; GLU, glucose; TRI, triglycerides; CREA, creatinine; ALT, alanine aminotransferase; AST, aspartate aminotransferase; BHB, β-hydroxybutyrate.

2 Values are expressed as means ± SEM.

In the context of the extreme environmental thermal load (93.17 THI vs. 56.37 THI baseline), an interaction effect was observed wherein both Harri and Naemi lambs showed significantly increased TP and ALB concentrations under heat stress (50 °C) compared to the thermoneutral baseline values of the Harri lambs (*p* = 0.043 and *p* = 0.046, respectively). Specifically, under heat stress, TP concentrations increased to 5.65 g/dL in Harri and 5.72 g/dL in Naemi lambs compared to the baseline Harri value of 5.03 g/dL. Similarly, ALB concentrations increased under heat stress to 2.71 g/dL in Harri and 2.69 g/dL in Naemi lambs relative to the baseline Harri value of 2.36 g/dL. These concentrations were not significantly affected by thermal transitions within the Naemi breed baseline values (5.28 g/dL TP and 2.54 g/dL ALB).

Additionally, exposure to extreme THI was associated with an interactive increase (*p* = 0.026) in TRI concentrations in both Harri and Naemi lambs under heat stress compared to the thermoneutral baseline value of Naemi lambs, while TRI values remained statistically unchanged from the baseline Harri values. Breed, temperature, or their interaction also exerted a significant effect on glucose (GLU) and urea concentrations (*p* < 0.05). Overall, Naemi lambs maintained lower circulating concentrations than Harri lambs for both GLU and urea (*p* < 0.05). Regarding environmental thermal treatments, the elevated THI corresponded with a significant main-effect increase in overall GLU (*p* = 0.047) and urea concentrations (*p* = 0.001). In contrast, breed, temperature, or their interaction had no significant effect (*p* > 0.05) on creatinine (CREA) concentrations.

Breed effect or the interaction between environmental thermal treatments (temperature) and breed showed a significant effect (*p* < 0.05) on alanine aminotransferase (ALT) activity, while ALT was not affected by temperature alone (*p* > 0.05). Naemi lambs maintained lower ALT activities across both environmental thermal treatments compared to Harri lambs (*p* = 0.035). Specifically, under baseline thermoneutral conditions, ALT activity was lower in Naemi lambs than in Harri lambs; this breed divergence persisted under acute heat stress, where Naemi lambs recorded an activity of 25.29 U/L compared to 28.25 U/L in Harri lambs. This indicates a baseline main breed effect (*p* = 0.002), with an overall mean activity of 25.37 U/L in Naemi versus 27.67 U/L in Harri lambs.

In contrast, the interaction between environmental thermal treatments (temperature) and breed had no effect on aspartate aminotransferase (AST) and β-hydroxybutyrate (BHB) concentrations (*p* > 0.05). However, AST was significantly modified by a standalone main breed effect (*p* = 0.024). Naemi lambs maintained a lower overall AST compared to Harri lambs, while transitioning from the thermoneutral baseline to extreme thermal exposure did not significantly alter overall AST activity (*p* = 0.783).

Regarding circulating ketone bodies, BHB concentrations were significantly influenced by both temperature and breed main effects (*p* < 0.05). Naemi lambs exhibited a lower overall mean BHB concentration relative to Harri lambs (*p* = 0.003). In line with the intense environmental thermal load, lambs under heat stress (50 °C) experienced a significant main-effect surge in BHB concentrations, increasing from an overall thermoneutral baseline of 0.32 mmoL/L up to 0.43 mmoL/L under extreme hyperthermia (*p* = 0.0001). At the individual level, this thermal shift provoked an increase in Harri lambs from 0.34 to 0.45 mmoL/L and in Naemi lambs from 0.31 to 0.42 mmoL/L.

### 3.4 Relative mRNA expression of target protective and metabolic genes

The effects of environmental thermal treatments (thermoneutral and acute heat stress) on the relative mRNA expression of protective and metabolic genes in hepatic tissue of Naemi and Harri lambs are presented in Table 6. The interaction between environmental thermal treatments (temperature) and breed had no significant effect on the relative mRNA expression values for any evaluated gene (*p* > 0.05), except for scavenger receptor class B member 1 (*SCARB1*), which was significantly affected (*p* = 0.038). Harri lambs showed higher relative expression of *SCARB1* under heat stress (50 °C) than under thermoneutral conditions and compared to Naemi lambs under both environmental treatments. Regarding breed effect, Naemi lambs had higher overall relative mRNA abundances for heat shock protein 90 (*HSP90*) and fibrinogen alpha chain (*FGA*) than Harri lambs across both environmental treatments (*p* = 0.045 and *p* = 0.049, respectively). In contrast, breed had no significant effect on the relative expression of heat shock protein 70 (*HSP70*), pyruvate kinase muscle isozyme 2 (*PKM2*), or *SCARB1* (*p* > 0.05). For environmental thermal treatments, heat stress (50 °C) was associated with higher relative expression of *HSP70*, *HSP90*, *PKM2*, *SCARB1*, and *FGA* than thermoneutral conditions at 25 °C (*p* < 0.05).

**Table 6:** Relative mRNA expression of hepatic protective and metabolic genes under thermoneutral and heat stress conditions in Naemi and Harri lambs.

Variables^1^		*HSP70*	*HSP90*	*PKM2*	*SCARB1*	*FGA*
Interaction (breed × temperature)						
Harri	25 °C	0.95	0.81	0.82	0.88^c^	0.96
	50 °C	1.49	1.32	1.20	1.37^a^	1.45
Naemi	25 °C	0.96	1.08	1.06	1.08^b^	1.09
	50 °C	1.88	1.46	1.21	1.19^b^	1.79
SEM^2^		0.28	0.10	0.09	0.08	0.11
*p*		0.502	0.518	0.220	0.038	0.352
Breed						
Harri		1.22	1.07^b^	1.01	1.12	1.21^b^
Naemi		1.42	1.27^a^	1.14	1.14	1.44^a^
SEM		0.19	0.07	0.06	0.06	0.07
*p*		0.480	0.045	0.198	0.862	0.049
Temperature						
25 °C		0.96^b^	0.95^b^	0.94^b^	0.98^b^	1.03^b^
50 °C		1.68^a^	1.39^a^	1.20^a^	1.28^a^	1.63^a^
SEM		0.19	0.07	0.07	0.06	0.08
*p*		0.015	0.002	0.012	0.001	0.0001

Different lowercase letters (a–c) within the same column indicate significant differences (*p* < 0.05) between temperature treatments or between breeds.

Data represent relative mRNA transcript abundance only. Because post-transcriptional regulation, translational delays, and protein turnover rates can decouple mRNA from functional protein, these data should not be used to infer absolute protein expression, enzyme kinetics, or active cellular protection.

1 Variables: *HSP70*, heat shock protein 70; *HSP90*, heat shock protein 90; *PKM2*, pyruvate kinase muscle isozyme2; *SCARB1*, scavenger receptor class B member 1; *FGA*, fibrinogen alpha chain.

2 Values are expressed as means ± SEM.

The effects of environmental thermal treatments (thermoneutral and acute heat stress) on the relative mRNA expression of protective and metabolic genes in the skeletal muscle tissue of Naemi and Harri lambs are presented in Table 7. The interaction between environmental thermal treatments (temperature) and breed had no significant effect on the relative mRNA expression values for any evaluated gene (*p* > 0.05), except for *SCARB1*, which was significantly affected (*p* = 0.002). Both Harri and Naemi lambs showed higher relative expression of *SCARB1* under heat stress (50 °C) than under thermoneutral conditions. For breed effect, Naemi lambs had higher overall relative mRNA abundances for *HSP70* and *PKM2* than Harri lambs across both environmental treatments (*p* = 0.040 and *p* = 0.042, respectively). In contrast, breed had no significant effect on the relative expression of *HSP90*, *SCARB1*, or *FGA* (*p* > 0.05). For environmental thermal treatments, heat stress (50 °C) was associated with higher relative expression of *HSP70*, *HSP90*, *PKM2*, and *SCARB1* than thermoneutral conditions at 25 °C (*p* < 0.05). The relative expression of *FGA* was not affected by temperature (*p* > 0.05).

**Table 7:** Relative mRNA expression of muscular protective and metabolic genes in the skeletal muscle tissue under thermoneutral and heat stress conditions in Naemi and Harri lambs.

Variables^1^		*HSP70*	*HSP90*	*PKM2*	*SCARB1*	*FGA*
Interaction (breed × temperature)						
Harri	25 °C	0.98	1.08	0.75	0.74^c^	1.15
	50 °C	1.56	1.38	1.82	1.64^a^	1.18
Naemi	25 °C	1.07	0.97	0.98	1.08^b^	1.07
	50 °C	1.85	1.72	1.96	1.45^a^	1.36
SEM^2^		0.12	0.12	0.12	0.08	0.11
*p*		0.432	0.072	0.724	0.002	0.286
Breed						
Harri		1.27^b^	1.23	1.28^b^	1.19	1.17
Naemi		1.46^a^	1.34	1.47^a^	1.27	1.22
SEM		0.09	0.08	0.08	0.06	0.08
*p*		0.040	0.344	0.042	0.367	0.683
Temperature						
25 °C		1.02^b^	1.02^b^	0.86^b^	0.91^b^	1.11
50 °C		1.71^a^	1.55^a^	1.89^a^	1.54^a^	1.27
SEM		0.08	0.08	0.09	0.05	0.08
*p*		0.0001	0.0001	0.0001	0.0001	0.184

Different lowercase letters (a–c) within the same column indicate significant differences (*p* < 0.05) between temperature treatments or between breeds.

Data represent relative mRNA transcript abundance only. Because post-transcriptional regulation, translational delays, and protein turnover rates can decouple mRNA from functional protein, these data should not be used to infer absolute protein expression, enzyme kinetics, or active cellular protection.

1 Variables: *HSP70*, heat shock protein 70; *HSP90*, heat shock protein 90; *PKM2*, pyruvate kinase muscle isozyme2; *SCARB1*, scavenger receptor class B member 1; *FGA*, fibrinogen alpha chain.

2 Values are expressed as means ± SEM.

The effects of environmental thermal treatments (thermoneutral and acute heat stress) on the relative mRNA expression of protective and metabolic genes in the adipose tissue of Naemi and Harri lambs are presented in Table 8. The results indicate that neither breed nor the interaction between environmental thermal treatments (temperature) and breed affected the relative expression of *HSP70*, *HSP90*, *PKM2*, and *SCARB1* (*p* > 0.05). In contrast, heat stress (50 °C) was associated with higher relative expression of *HSP70*, *HSP90*, *PKM2*, and *SCARB1* compared to thermoneutral conditions at 25 °C (*p* < 0.05). The interaction between temperature and breed significantly affected the relative expression of *FGA* (*p* = 0.0001). Naemi lambs showed higher relative expression of *FGA* under heat stress (50 °C) than under other treatments. Overall, Naemi lambs had higher relative expression of *FGA* than Harri lambs (*p* = 0.0001). For environmental thermal treatments, heat stress (50 °C) was associated with higher relative expression of *FGA* than thermoneutral conditions at 25 °C (*p* = 0.0001).

**Table 8:** Relative mRNA expression of adipose protective and metabolic genes in adipose tissue under thermoneutral and heat stress conditions in Naemi and Harri lambs.

Variables^1^		*HSP70*	*HSP90*	*PKM2*	*SCARB1*	*FGA*
Interaction (breed × temperature)						
Harri	25 °C	0.87	0.98	0.96	0.91	1.12^b^
	50 °C	1.51	1.43	1.63	1.83	1.29^b^
Naemi	25 °C	1.05	1.10	1.12	1.02	1.26^b^
	50 °C	1.53	1.61	1.70	1.89	2.19^a^
SEM^2^		0.11	0.11	0.16	0.07	0.06
*p*		0.471	0.815	0.780	0.758	0.0001
Breed						
Harri		1.19	1.21	1.30	1.37	1.21^b^
Naemi		1.29	1.35	1.41	1.45	1.73^a^
SEM		0.07	0.08	0.11	0.05	0.05
*p*		0.349	0.210	0.491	0.291	0.0001
Temperature						
25 °C		0.96^b^	1.04^b^	1.04^b^	0.96^b^	1.19^b^
50 °C		1.52^a^	1.52^a^	1.67^a^	1.87^a^	1.74^a^
SEM		0.08	0.07	0.11	0.06	0.04
*p*		0.0001	0.002	0.005	0.0001	0.0001

Different lowercase letters (a–b) within the same column indicate significant differences (*p* < 0.05) between temperature treatments or between breeds.

Data represent relative mRNA transcript abundance only. Because post-transcriptional regulation, translational delays, and protein turnover rates can decouple mRNA from functional protein, these data should not be used to infer absolute protein expression, enzyme kinetics, or active cellular protection.

1 Variables: *HSP70*, heat. shock. protein 70; *HSP90*, heat. shock. protein 90; *PKM2*, pyruvate kinase muscle isozyme2; *SCARB1*, scavenger receptor class B member 1; *FGA*, fibrinogen alpha chain.

2 Values are expressed as means ± SEM.

## 4 Discussion

Successfully executing a controlled hyperthermic challenge requires rigorous verification that the experimental microclimate transitions from a true baseline state to a severe, uniform thermal load without exceeding the absolute physiological limits of the biological models (25, 26). In this research, this homeostatic boundary was maintained, as indicated by the absence of significant mortality in both the Naemi and Harri lamb populations during the acute 180-min exposure period. This survival rate demonstrates that, although the artificial microclimate was extremely challenging, it remained within a sublethal range. This allows for the characterization of active physiological and transcriptional changes rather than irreversible pathological system failure or terminal heat stroke, which can occur during uncontrolled hyperthermic extremes in non-adapted small ruminants (19, 27).

The microclimatic variations observed between the experimental phases confirm that the programmed simulation profiles produced a highly distinct environmental transition. During the baseline phase, both breed cohorts were kept under stable thermoneutral conditions (24.63 °C Ta, 38.0% RH, 56.37 THI). According to established ruminant bioclimatology frameworks, a thermal load within this baseline range (THI < 70) indicates a complete absence of thermal stress, confirming that the lambs were in ideal homeostatic equilibrium before the challenge (28, 29). In contrast, the transition to the acute hyperthermic stage caused an extreme shift in the chamber microclimate, with the temperature reaching 51.15 °C and 13.22% RH. This shift pushed the calculated THI to an extreme value of 93.17, far exceeding the traditional threshold for severe heat stress (THI > 82) in small ruminants and matching values known to trigger profound cellular and systemic stress responses (30). However, the uniform drop in relative humidity and the corresponding extreme surges in ambient temperature and thermal index across both groups confirm that the Naemi and Harri cohorts were exposed to an identical, standardized hyperthermic environment. This clear environmental distinction between the thermoneutral baseline and the heat stress challenge provides a highly reliable foundation for interpreting subsequent breed-specific physiological trade-offs and transcript-level responses without confounding microclimatic biases, consistent with the methodology required for robust climate-controlled animal phenotyping (31).

The within-breed before-after changes observed for RR, Tsk, Tct, and AC are consistent with thermal regulation in these breeds reflecting integrated physiological trade-offs rather than uniform, parallel shifts. The surge in RR under acute hyperthermia represents a classic small ruminant defense mechanism, using rapid movement of the upper respiratory tract to drive respiratory evaporative heat dissipation (32). Because the thermal conductivity of the environment at extreme ambient temperatures (50 °C) exceeds normal body surface temperatures, sensible heat loss pathways (conduction, convection, and radiation) become entirely ineffective, leaving evaporative cooling as the sole viable method for shedding heat (33).

The substantial investment in panting mechanics by the Harri lambs (increasing from 41.27 to 120.34 breaths/min) protected their core body temperature baseline under the interactive model (39.33 °C vs. 39.84 °C). However, this active homeothermic defense imposes a significant metabolic cost, as sustained hyperventilation requires continuous energy expenditure to power the respiratory musculature (34). In contrast, the Naemi breed exhibited a more resource-conserving strategy based on transient heterothermy. Rather than expending immediate metabolic reserves on intense respiratory cooling to defend a rigid internal core baseline, Naemi lambs tolerated a temporary rise in rectal temperature (38.91 °C to 39.76 °C) combined with enhanced coat surface thermal dissipation. These divergent thermoregulatory responses highlight distinct physiological trade-offs between energetic expenditure and core temperature maintenance, rather than reflecting differences in intrinsic breed superiority. This is consistent with studies suggesting that peripheral cooling mechanisms play a dominant role in thermoregulation in heat-adapted breeds (35). The larger increase in Tct in Naemi sheep may also indicate differences in coat properties that influence heat absorption and reflection, allowing for more effective heat exchange with the environment (36). These findings show that the two breeds rely on divergent thermoregulatory strategies with distinct physiological trade-offs: the Harri breed defends its core temperature through high-cost respiratory mechanics, whereas the Naemi breed relies on transient heterothermy and greater coat conductance, consistent with established physiological profiles of desert-adapted ruminants though the direct welfare implications of this strategy remain to be fully characterized using validated behavioral indicators (11).

The changes in serum biochemical and hepatic enzyme responses indicate systemic homeostatic adjustments made by the two breeds to support their distinct whole-animal thermoregulatory strategies (1). The stable serum CREA concentration across all experimental groups shows that, despite the extreme thermal load, neither population experienced significant renal impairment or severe dehydration, which often disrupts clearance mechanisms during uncompensated heat strain (37). The overall increase in circulating GLU and BHB during acute hyperthermia suggests accelerated mobilization of readily available energy stores to meet the immediate, high-priority demands of thermoregulatory processes, such as respiratory muscle activity (38). The increased TRI and BHB in Harri lambs under heat stress may result from active reliance on adipose reserve mobilization and subsequent hepatic ketogenesis (39). This metabolic shift provides essential fuel to sustain their rapid, energy-intensive panting mechanism, but it imposes a high systemic cost (20). In contrast, Naemi lambs resisted this hyperthermic ketogenesis, maintaining TRI and BHB levels close to initial baselines, consistent with a heterothermic strategy that limits reliance on ketogenic fuel mobilization during acute heat stress (40).

The observed increase in TP and ALB concentrations under acute heat stress is a classic physiological marker of acute plasma volume contraction and hemoconcentration, a finding that aligns with observations in other desert-adapted small ruminants exposed to severe thermal loads (41). This phenomenon typically results from substantial evaporative moisture loss during sustained tachypnea. Likewise, the elevation of serum urea under extreme THI reflects a well-documented physiological strategy in indigenous sheep to conserve nitrogen and recycle urea to the rumen when internal water availability is limited, thereby maximizing metabolic water retention (42). When comparing breeds, the lower baseline GLU, urea, and BHB levels maintained by Naemi lambs across both environments suggest a distinct baseline metabolic profile relative to Harri lambs, reflecting a different physiological strategy rather than a superior one. This pattern is broadly consistent with comparative studies of other desert-adapted breeds (e.g., Awassi or Malpura) relative to temperate breeds, where arid-adapted animals exhibit moderated metabolic expenditures under acute challenges (6). Conversely, the marked elevation of glucose, triglycerides, and BHB in Harri lambs under heat stress is consistent with an energy-demanding homeothermic defense driven by high-cost respiratory exertion.

Regarding cellular and hepatic integrity, the significant interaction affecting ALT activity, contrasted with the absence of an interactive change for AST, provides important insight into cellular stress boundaries. The stable circulating levels of AST confirm that the 180-min acute exposure did not induce profound hepatic necrosis or irreversible muscular cellular injury, which is consistent with the lack of animal mortality (43). However, the consistently lower hepatic enzymatic activities (ALT and AST) in Naemi lambs compared with Harri lambs may reflect comparatively lower enzymatic leakage under this acute exposure, without demonstrating greater long-term cellular resilience or thermotolerance.

These physiological comparisons provide critical insights for developing tailored heat stress mitigation strategies across low-input farming systems in hyper-arid regions. Because Harri lambs rely heavily on an energy-intensive, water-depleting respiratory cooling mechanism (panting), farmers raising this breed under extreme summer conditions, particularly where mechanical cooling is not feasible, should consider active mitigation measures, including supplementing diets with high-quality bypass fats or fermentable energy sources to replenish the substantial glucose and triglyceride depletion observed during acute heat stress, without increasing dietary crude protein, which could exacerbate urea accumulation. They should also use intensive shaded housing, forced ventilation, or evaporative cooling misters during peak THI periods to artificially lower the ambient heat load and prevent elevated TP and ALB in this high-exertion breed. These management implications reflect the distinct short-term coping strategy identified in Harri lambs under this severe acute challenge and should not be interpreted as evidence that the Naemi breed is inherently superior, since long-term productivity, welfare, and survival outcomes were not assessed in this study. It is important to emphasize that these management suggestions are derived from responses to a provocative, 3-h acute 50 °C challenge rather than the cyclic, diurnal thermal patterns typical of commercial production systems. As such, they should be regarded as reflecting acute emergency coping responses rather than chronic or field-representative heat-stress conditions, and their practical value for farmers remains to be confirmed through validation trials conducted under real on-farm heat-stress conditions before they are adopted as standing management recommendations.

The multi-tissue transcriptomic profiles analyzed in the liver, skeletal muscle, and adipose tissues reveal a highly coordinated, tissue-specific molecular transcriptional network that supports the whole-animal acute response profiles of both lamb breeds. The universal upregulation of heat shock proteins transcripts (*HSP70* and *HSP90*) across hepatic, muscular, and adipose tissues under heat stress represents a candidate transcript-level response associated with heat-induced proteotoxicity. To mitigate this structural stress, tissues rapidly increase the relative abundance of transcripts encoding molecular chaperones as an immediate transcriptional response (12, 44). The higher relative expression of *HSP90* transcripts in the liver of Naemi lambs, along with their higher relative expression of *HSP70* transcripts in muscle, reflects a proactive transcriptional readiness, though downstream translation into functional chaperone proteins or actual cellular protection remains to be verified (45, 46). In contrast, the lower relative mRNA expressions in Harri lambs may suggest a physiological approach focused on external heat dissipation rather than internal tissue transcriptional activation.

Our results showed that the relative expression of *PKM2* was higher in all evaluated tissue matrices under heat stress. Pyruvate kinase is a key regulatory enzyme in the final step of the glycolytic pathway, controlling the conversion of phosphoenolpyruvate to pyruvate with the concurrent generation of ATP (47). Under heat stress, cellular oxygen delivery is often compromised due to shifts in peripheral vascular perfusion, creating localized hypoxic or energy-depleted microenvironments (48). The significantly higher baseline and hyperthermic abundance of *PKM2* transcripts observed in the muscle of Naemi lambs suggests an active glycolytic priming at the gene expression level, though enzyme activity assays are required to confirm shifts in ATP generation pathways (49).

The interaction patterns observed for relative *SCARB1* expression reveal highly specialized, breed-dependent adjustments in lipid mobilization and trafficking. The *SCARB1* protein plays a critical role in cellular cholesterol uptake and high-density lipoprotein metabolism, serving as a key regulator of lipid transport across cellular membranes during heat stress (50). Analysis of the interaction term shows that Harri lambs markedly upregulated *SCARB1* expression in both hepatic and skeletal muscle tissues under heat stress. This molecular increase in the Harri breed directly coincides with their systemic biochemical profile, aligning with their significant hyperthermic rise in circulating TRI and BHB. Collectively, these findings suggest that Harri lambs may activate a specialized molecular pathway to accelerate intracellular transport and catabolism of fat reserves, providing an essential lipid fuel source to sustain their energy-intensive panting mechanism (51). As with the other target genes, this interpretation reflects a transcript-level association only; SCARB1 mRNA abundance does not confirm changes in SCARB1 protein expression, membrane trafficking, or lipid-transport activity, which would require protein-level validation. The tissue-specific interaction influencing *FGA* expression in adipose tissue offers new descriptive insight into the genomic responses of desert ruminants, though its exact biological role remains to be elucidated. Fibrinogen alpha chain (*FGA*) is traditionally recognized as an acute-phase reactant involved in systemic inflammation, coagulation, and tissue repair (52). While hepatic *FGA* transcripts were universally upregulated across both breeds to support the classic systemic acute-phase response (53), the massive, localized surge in *FGA* transcripts within the subcutaneous adipose tissue of Naemi lambs points to a highly localized, breed-specific genomic mobilization. In the absence of corresponding extracellular matrix characterization, adipocyte histomorphology, local fibrosis assessments, or validated protein-level expression data, the functional relevance of this tissue-specific transcript surge cannot be determined. Whether this expression pattern reflects an adaptive structural response, a localized acute-phase reaction, or an uncoupled transcriptional artifact remains an open hypothesis requiring comprehensive downstream validation.

The extreme nature of the 3-h acute exposure (50 °C, 13% RH) used in this trial warrants critical evaluation of its generalizability to routine commercial production. In standard production systems, lambs typically experience cyclic, diurnal thermal patterns with lower peak temperatures and variable humidity, rather than an abrupt, constant hyperthermic shock. Consequently, the immediate physiological changes and tissue-specific molecular transcriptional shifts documented here represent an acute emergency coping profile rather than an acclimated baseline state. This challenge model was intentionally selected as a robust diagnostic environment to push homeostatic boundaries to their upper thresholds, effectively uncoupling breed-specific genetic signatures from confounding farm-level variables. Despite its severity, this experimental design has direct ecological and practical relevance for commercial animal management in arid and semi-arid regions. As global climate volatility increases, commercial herds are increasingly exposed to extreme meteorological events and sudden-onset desert heatwaves in which microclimatic indexes can briefly match these severe thresholds. Mapping acute thresholds helps establish accurate engineering parameters for forced ventilation and crisis-cooling protocols in commercial infrastructure. Furthermore, identifying the specific transcriptional responses and candidate genes that vary between breeds under acute exposure provides baseline molecular targets for future functional validation studies.

While these highly synchronized transcriptomic shifts provide a clear view of the immediate cellular response to acute hyperthermia, a notable limitation of the current investigation is the absence of parallel protein-level validation (such as Western blotting, ELISA, or immunohistochemistry) as well as direct endocrine (e.g., cortisol, ACTH) and oxidative stress markers. Post-transcriptional regulation, translational delays, and varying protein turnover rates can decouple mRNA abundance from absolute functional protein expression. For instance, the massive localized surge in *FGA* or *SCARB1* transcripts indicates active genomic mobilization but does not definitively confirm an equivalent increase in functional scaffolding or lipid transport proteins at the cell surface. Consequently, these multi-tissue profiles should be interpreted as primary transcriptomic signatures of heat shock, and future work incorporating quantitative proteomic, neuroendocrine, and redox-status assays is required to verify that these acute transcriptional shifts successfully translate into functional protein activity and systemic homeostatic stability.

## 5 Conclusion

This research indicates that indigenous desert-adapted small ruminants use highly divergent physiological trade-offs when confronted with severe, acute hyperthermic challenges. The empirical findings support our initial hypothesis, confirming that macro-physiological adjustments and micro-molecular transcriptional responses are closely integrated in a breed-dependent manner. Harri lambs prioritize an aggressive, immediate homeothermic defense, relying heavily on energy-intensive respiratory cooling (panting) to maintain internal core temperature stability under acute thermal stress. This intense physical effort imposes significant metabolic strain, directly accompanied at the biochemical and tissue levels by rapid plasma volume contraction, accelerated hepatic ketogenesis, and targeted upregulation of multi-tissue *SCARB1* transcripts. In contrast, Naemi lambs show a pattern consistent with acute heat buffering at the transcript level, reflecting a distinct energy-conserving physiological strategy. They employ transient heterothermy – safely tolerating a controlled rise in core temperature – supported by greater coat heat conductance and a baseline metabolic profile that limits systemic ketogenesis. At the cellular level, this tolerance is characterized by elevated baseline transcripts of hepatic *HSP90*, muscular *HSP70*, and metabolic *PKM2*. A key finding is the localized surge of *FGA* transcripts within the fat tail of Naemi lambs, revealing a tissue-specific transcriptional marker whose downstream protective translation requires functional validation.

In conclusion, although both breeds are robust under hyper-arid extremes, Harri and Naemi lambs exhibit divergent short-term physiological and transcriptional coping strategies with distinct metabolic trade-offs. Naemi lambs show a pronounced transcript-level buffering signature and a distinct fat-tail mRNA response; these findings should be interpreted as candidate molecular associations pending protein-level validation and do not, on their own, indicate superior long-term productivity, welfare, or survival for either breed. Ultimately, while this study successfully characterizes the divergent transcriptional architectures of these indigenous breeds under severe thermal stress, future proteomic profiling, along with endocrine and oxidative stress characterization, remains essential to definitively validate the translation of these molecular mechanisms into functional systemic resilience. Because the thermoneutral and heat-stress conditions were administered in a fixed, non-randomized order, the breed × temperature interactions summarized above are best understood as breed-dependent, within-breed before-after differences rather than as independent causal effects of temperature. More broadly, because the 50 °C exposure used here represents a provocative diagnostic model designed to isolate homeostatic limits rather than typical production conditions, all findings reported in this study should be understood as characterizing acute emergency physiological and transcriptional responses. Extrapolation of these findings to routine field or commercial management practices should await validation under representative on-farm heat-stress conditions before specific practical recommendations are made for farmers.

## Data Availability

The original contributions presented in the study are included in the article/supplementary material, further inquiries can be directed to the corresponding author.
